# Bilateral Burkitt Lymphoma of the Ovaries: A Report of a Case in a Child with Williams Syndrome

**DOI:** 10.1155/2011/327263

**Published:** 2011-05-26

**Authors:** Grace Ifeyinwa Onimoe, Samir Kahwash, Amanda Termuhlen, Thomas G. Gross, Elizabeth Varga, Melissa J. Rose

**Affiliations:** ^1^Department of Pediatrics, College of Medicine, The Ohio State University, Columbus, OH 43210, USA; ^2^Department of Pediatric Hematology-Oncology, Nationwide Children' Hospital, Columbus, OH 43205, USA; ^3^Department of Pathology, College of Medicine, The Ohio State University, Columbus, OH 43210, USA

## Abstract

A 10-year-old female with Williams Syndrome (WS) presented with a two-month history of fatigue, weight loss, and bilateral ovarian masses. Histologic, immunophenotypic, and cytogenetic studies confirmed the diagnosis of Burkitt lymphoma (BL). While there is no established association between the two disorders, this is the third case in the literature of Burkitt lymphoma in a patient with Williams Syndrome.

## 1. Introduction

Although relatively rare in adults, Burkitt lymphoma constitutes more than 40% of pediatric non-Hodgkin lymphomas (NHL). The World Health Organization (WHO) classification (2008) recognizes three clinical subtypes: endemic, sporadic, and immunodeficiency associated. In the Western Hemisphere, most cases present in the gastrointestinal tract. Cases involving the jaw, kidneys, breasts, and other sites are common, including bilateral involvement [1].

Williams Syndrome (also known as Williams-Beuren Syndrome) is a rare genetic disorder with an incidence of one per 7,500–20,000 births; most cases occurring sporadically. Clinical characteristics of Williams Syndrome are growth and mental retardation with a friendly, outgoing personality, dysmorphic facial features, hypercalcemia in early infancy, and congenital cardiovascular malformations, in particular supravalvular aortic stenosis [2]. Williams Syndrome is the consequence of an interstitial microdeletion at 7q11.23, which includes the elastin gene producing hemizygosity at the elastin gene locus.

We describe the third case of Burkitt lymphoma in a child with Williams Syndrome [3, 4].

## 2. Case Presentation

A 10-year-old female diagnosed with Williams Syndrome at age 9 years, confirmed by fluorescent in situ hybridization (FISH), presented with a two-month history of fatigue, decreased activity, weight loss, regression in bladder control, and a recently palpated pelvic mass. Her medical history was also significant for congenital hypothyroidism and aortic valvular stenosis. 

Physical examination showed WS facial dysmorphia, murmur consistent with aortic valvular stenosis, mild hypertension, and a 2.5 cm by 2.5 cm protruding mass just medial to the left anterior superior iliac spine, without defined edges. 

**Figure 1:**
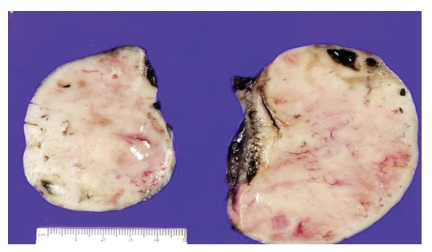
Sectioned right and left ovaries, with near-total obliteration of the usual follicular cut surface, replaced by the typical tan white appearance of lymphoma.

**Figure 2:**
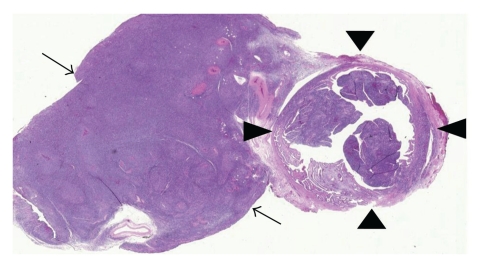
Low power photomicrograph showing sheets of lymphoma cell replacing the usual histology of ovary (arrow) and infiltrating fallopian tube (arrow heads).

Abdominal CT study revealed four discrete masses: two large masses within the pelvis causing bilateral hydronephrosis, a third involving the hilum of the left kidney interfering with arterial and venous blood flow of the kidney, and a fourth involving the head of the pancreas. Metastasis to the left femoral diaphyses and proximal left humerus was also noted. Laboratory evaluation demonstrated a progressively elevated LDH, normal uric acid, BUN, and creatinine. Serological workup for Epstein Barr Virus and cytomegalovirus was negative. The patient underwent exploratory laparotomy, salpingo-oophorectomy, partial omentectomy, and a wedge biopsy of right lobe of liver. The cut surface of the ovaries is shown in Figure 1. Microscopic examination of the ovaries showed diffuse infiltrates consisting of medium-sized hyperchromatic cells with irregular nuclei, and small multiple nucleoli (Figure 2). Mitotic activity was high, and there were areas of increased debris-laden macrophages, giving the appearance of a “starry-sky”. Cell marker study by flow cytometry revealed a monotypic lymphoid cell population, positive for CD10, CD19, CD20, and HLA: DR with kappa light chain restriction. Cytogenetics and molecular genetics showed 46, XX, t(8; 14) (q24; q32).

There was no evidence of tumor in the liver or omentum. The bone marrow and CSF were negative for malignant cells. The results of pathology and staging indicated Stage III Burkitt lymphoma (by Murphy staging) [5]. 

Intention was to treat the patient as per FAB/LMB96 regimen B4, but therapy reductions were made due to complications [6]. Her regimen included COP (cyclophosphamide 300 mg/m^2^, vincristine 1 mg/m^2^ once, prednisolone 60 mg/m^2^/day for 7 days, and intrathecal chemotherapy with methotrexate/hydrocortisone (once)), COPADM1 (cyclophosphamide 250 mg/m^2^ BID for 6 doses, vincristine 2 mg/m^2^ once, prednisolone 60 mg/m^2^/day for 5 days, then taper over 3 days, doxorubicin 60 mg/m^2^ once, methotrexate 3000 mg/m^2^ once, and methotrexate/hydrocortisone given intrathecally twice), dose-reduced COPADM2 (cyclophosphamide 250 mg/m^2^ BID for 6 doses, vincristine 1 mg/m^2^ once, prednisolone 60 mg/m^2^/day for 5 days, then taper over 3 days, doxorubicin 30 mg/m^2^ once, methotrexate 1500 mg/m^2^ once, and methotrexate/hydrocortisone given intrathecally twice), and dose-reduced CYM1 (cytarabine 100 mg/m^2^ continuous infusion daily for 5 days, methotrexate 1500 mg/m^2^ once, and intrathecal methotrexate/hydrocortisone once and cytarabine/hydrocortisone once). The last cycle of chemotherapy, CYM2, was omitted. Rituximab was given every 2–4 weeks for 5 total doses, starting between COPADM1 and COPADM2, with a cumulative dose of 1875 mg/m^2^. 

Our patient developed several complications during and after therapy. These included hypertension and renal insufficiency requiring intensive care. She presented with a small bowel obstruction after induction therapy, followed by abdominal compartment Syndrome; both required surgical intervention and prolonged administration of parental nutrition. She experienced a two-month delay in chemotherapy to allow for healing. The patient had multiple episodes of febrile neutropenia with concomitant sepsis producing delays and dose reduction of chemotherapy. Chemotherapy was discontinued early due to poor tolerance. Cytopenias persisted for several months off chemotherapy, but did not require intervention with transfusions or growth factors and spontaneously resolved at approximately 6 months after rituximab completion. At the time of this report, she has been off therapy for 22 months and remains in complete remission.

## 3. Discussion

Features of WS include congenital heart disease, hypertension, premature aging of skin, dysmorphic facial features, infantile hypercalcemia, gregarious personality, and mental retardation with Intelligence Quotients ranging from 20 to 106 [7]. A submicroscopic deletion of contiguous genes on the long arm of chromosome 7 is seen in Williams Syndrome. Chromosome 7 is the second most frequently involved chromosome among the cytogenetic abnormalities observed in human malignancies after chromosome 8 [8]. The cytogenetic abnormalities in human cells are considered acquired; however, cancer has been reported in persons with constitutional cytogenetic abnormalities involving chromosome 7 [9–15], and it has been speculated that these abnormalities may predispose to malignancies. The literature contains reports of malignancy occurring in Williams Syndrome including five cases in the pediatric age group and four cases in the adult age group [16]. The pediatric cases include two cases of Burkitt lymphoma (ages 1 and 5 years), a Cutaneous fibrous harmartoma (age 5 years), acute lymphoblastic lymphoma (age 14 years), and astrocytoma in a 5-year-old child [16]. 

The two prior reported patients with WS and BL tolerated chemotherapy well, without unexpected complications; one patient received standard-dosing chemotherapy per the same protocol as our patient, but followed the B1 arm which included the second CYM cycle as well as COPADM3, while the other patient received 4 courses of CHOP (cyclophosphamide, doxorubicin, vincristine and, prednisolone). Due to the inability to deliver full intensity chemotherapy, treatment was modified in our patient, by adding rituximab, reducing doses of cyclophosphamide, methotrexate, doxorubicin, and vincristine, and deleting the second cycle of consolidation therapy. Though the efficacy of rituximab in BL has yet to be proven, there is data showing that rituximab has activity in pediatric Burkitt lymphoma [17]. The efficacy of rituximab is difficult to determine for this patient. Our patient's medical complications were likely multifactorial in etiology and may or may not be related to her underlying WS. It is important to note that so far, there is no established association between the genetic consequences of the chromosomal abnormalities observed in WS and BL. 

Most patients with WS have a *de novo* submicroscopic deletion of 7q11.23 detectable by FISH [2, 4, 7]. WS is a contiguous gene Syndrome caused by 1.5 megabase microdeletion, which is most often due to unbalanced recombination during meiosis. The critical region encodes the elastin gene (*ELN*), which contributes to supravalvular aortic stenosis, hoarse voice, and some of the characteristic facial features of WS, and there are at least 25 other genes adjacent to the *ELN* gene that may also contribute to the phenotype [2, 18–20]. A number of these genes have been described as being involved in mediating processes that can lead to the development of malignancy. A review of the literature shows that one gene (*BAZ1B*) also known as Williams-Beuren Syndrome transcription factor (*WSTF*) is associated with response/repair to DNA damage [21]. Xiao et al. demonstrated that WSTF has intrinsic tyrosine kinase activity which allows it to phosphorylate H2A.X, a specialized histone variant. This prompts chromatin remodeling necessary for DNA repair. Inability to repair DNA leads to genomic instability, carcinogenesis, and cell death. This pathway is of possible interest since H2A.X deficiency has been shown to accelerate B and T cell lymphoma development in p53 deficient mice [22, 23]. Further, H2A.X-deficient mouse embryonic fibroblasts and B and T cells display pronounced levels of genomic instability [24]. Another gene, *LIMK1,* deleted in the WS critical region has been associated with prostate cancer metastasis [25]. A third gene named *GTF2IRD1* interacts with the retinoblastoma (*RB1*) gene *in vitro* and *in vivo* [26]. *RB1* is a known regulator of cell cycle and development, as well as an important tumor suppressor. 

Finally, the *BCL7* gene family is of greatest interest in association with BL. The *BCL7A* gene maps to chromosome 12 and occurs in translocation with a Burkitt lymphoma cell line [27]. The *BCL7B *gene maps within the commonly deleted Williams Syndrome region [28], and 90 percent identify with the N-terminal 51 amino acids of *BCL7A *but investigators have not implicated it in cancer so far. Whether the hemizygosity of BCL-7B disrupts important developmental processes or is implicated in the pathogenesis of a lymphomatous proliferation remains to be answered [3]. 

 We, like others, speculate that the Williams Syndrome deletion could predispose to a malignant development through the loss of heterozygosity in a tumor suppressor gene, but more studies are necessary to establish a true association [3]. 

In conclusion, while there is no established link between WS and an increased incidence of malignancy or complications with chemotherapy, more case reports with detailed molecular findings are needed to facilitate exploring the possibility of a linkage.
